# Curso de maternidad y paternidad a gestantes, en contextos clínicos de Valledupar, Colombia

**DOI:** 10.15446/rsap.V27n4.120935

**Published:** 2025-07-01

**Authors:** Margareth Corzo-Contreras, Julia K. Mejía-Rincón, María C. Murgas-Quintero, Juan C. García-Ubaque, Belkis V. Cuesta-Morato

**Affiliations:** 1 MC: Enf. M. Sc. Salud Sexual y Reproductiva. Universidad Popular del Cesar. Valledupar, Colombia. margarethcorzo@unicesar.edu.co Universidad Popular del Cesar Salud Sexual y Reproductiva Universidad Popular del Cesar Valledupar Colombia margarethcorzo@unicesar.edu.co; 2 JM: Enf. M. Sc. Ciencias de la Enfermería. Universidad Popular del Cesar. Valledupar, Colombia. Juliamejia@unicesar.edu.co Universidad Popular del Cesar Ciencias de la Enfermería Universidad Popular del Cesar Valledupar Colombia Juliamejia@unicesar.edu.co; 3 MM: Enf. Esp. Cuidado Neonatal. M. Sc. Salud Pública. Universidad Popular del Cesar. Valledupar, Colombia. mariamurgas@unicesar.edu.co Universidad Popular del Cesar Salud Pública Universidad Popular del Cesar Valledupar Colombia mariamurgas@unicesar.edu.co; 4 JG: MD. Ph. D.; M. Sc. Salud Pública. Universidad Nacional de Colombia. Docente, Facultad de Medicina. Departamento de Salud Pública. Bogotá, Colombia. jcgarciau@unal.edu.co Universidad Nacional de Colombia Universidad Nacional de Colombia Facultad de Medicina Departamento de Salud Pública Bogotá Colombia jcgarciau@unal.edu.co; 5 BC: Enf. M. Sc. Salud Sexual y Reproductiva. Universidad Popular del Cesar. Valledupar, Colombia. belkiscuesta@unicesar.edu.co Universidad Popular del Cesar Salud Sexual y Reproductiva Universidad Popular del Cesar Valledupar Colombia belkiscuesta@unicesar.edu.co

**Keywords:** Educación prenatal, atención hospitalaria, parto humanizado, paternidad, percepción social *(fuente: DeCS, BIREME)*, Prenatal education, hospital care, humanized childbirth, parenthood, social perception *(source: MeSH, NLM)*

## Abstract

**Introducción:**

El acompañamiento a la maternidad es una estrategia que permite brindar educación oportuna a la pareja gestante, con el fin de facilitar la apropiación de habilidades para afrontar de manera segura los cambios físicos, emocionales y sociales propios del proceso gestacional. El escenario hospitalario puede ser una oportunidad desaprovechada para incentivar la adherencia a este tipo de cursos.

**Objetivo:**

Conocer las experiencias de las gestantes hospitalizadas en relación con la implementación de un curso de maternidad y paternidad.

**Metodología:**

Se realizó un estudio cualitativo fenomenológico descriptivo, en una población de 12 gestantes hospitalizadas en una IPS de la ciudad de Valledupar, utilizando entrevistas en profundidad. Se garantizó la validación interna y externa a través de la triangulación. Asimismo, se consideraron aspectos éticos fundamentales como la confidencialidad de los datos y el respeto por la dignidad de las participantes.

**Resultados:**

Las participantes, en su mayoría primigestantes, expresaron que la información recibida les permitió aclarar dudas y fortalecer conocimientos en temas como la lactancia materna, el parto humanizado y el autocuidado.

**Conclusión:**

Las participantes destacaron su impacto positivo en la preparación emocional y práctica para la maternidad, la corrección de mitos y la construcción de nuevos saberes. Si bien la experiencia fue valorada favorablemente, se evidenció la necesidad de mejorar su difusión y accesibilidad, especialmente en zonas rurales.

## Abstract (en)

**Introduction:**

Maternity support is a strategy that allows providing timely education to the pregnant couple to facilitate the appropriation of skills to safely face the physical, emotional and social changes of the gestational process. The hospital setting may be a missed opportunity to encourage adherence to this type of course.

**Objective:**

To learn about the experiences of hospitalized pregnant women in relation to the implementation of a maternity and paternity course.

**Methodology:**

A descriptive phenomenological qualitative study was conducted in a population of 12 pregnant women hospitalized in an IPS of the city of Valledupar using in-depth interviews. Internal and external validation was guaranteed through triangulation. Likewise, fundamental ethical aspects were considered, such as data confidentiality and respect for the dignity of the participants.

**Results:**

The participants, mostly primigestants, expressed that the information received allowed them to clarify doubts and strengthen their knowledge on topics such as breastfeeding, humanized childbirth and self-care.

**Conclusion:**

The participants highlighted its positive impact on the emotional and practical preparation for motherhood, the correction of myths and the construction of new knowledge. Although the experience was favorably evaluated, the need to improve its dissemination and accessibility, especially in rural areas, was evident.

A lo largo de la historia, el acompañamiento a la maternidad ha estado presente como una manifestación social y cultural del cuidado, dirigido tanto a la madre como al recién nacido. El avance en la ginecoobstetricia ha proporcionado evidentes e indiscutibles beneficios, por lo que acuerdos internacionales como los Objetivos de Desarrollo Sostenible y normas locales establecen estrategias orientadas a la protección de la mujer y del fruto de la gestación durante el embarazo, el parto y el puerperio, buscando así garantizar una vida sana, la disminución de la morbilidad y mortalidad materna y neonatal, la generación de impactos positivos en las conductas de salud de las gestantes y en, general, promoviendo el bienestar 1-3.

Sin embargo, la participación significativa en los programas educativos dirigidos a mujeres gestantes y sus familias -centrados en el embarazo, el trabajo de parto y el periodo posparto- sigue siendo un desafío; las realidades del entorno de muchas gestantes puede impedir que reciban atención adecuada durante su proceso reproductivo, ya sea por fallos del sistema de salud, desigualdad de género, trato irrespetuoso por parte del profesional sanitario, u otros determinantes que obstaculizan las intervenciones orientadas al bienestar del binomio madre-hijo 4.

Desde esta perspectiva, el enfoque de la salud materna no solo se centra en el objetivo de lograr prevenir la enfermedad y sus consecuencias, sino en el proceso integral que implica el reconocimiento pleno de los derechos sexuales y reproductivos, así como en el desarrollo de acciones orientadas a una maternidad segura, garantizando una atención adecuada, oportuna e integral durante la gestación, el parto y el puerperio, con base en principios de respeto, equidad, participación del personal de salud y acompañamiento del entorno familiar a lo largo de la experiencia reproductiva 4.

El enfoque de cuidado integral y respetuoso responde a necesidades clínicas y humanas inmediatas: todas las mujeres embarazadas tienen derecho a recibir una atención de calidad que garantice su vida y bienestar, ya que ninguna debería morir por causas prevenibles antes, durante o después del parto, por lo que se deben evitar las barreras económicas, geográficas o administrativas al tiempo que se gestionan las barreras culturales y se impulsa el empoderamiento de las mujeres y su derecho a acceder a servicios de salud sexual y reproductiva 3,5,6.

En Colombia se ha establecido la denominada Ruta Integral de Atención en Salud para la Población Materno Perinatal, normativa que busca garantizar un proceso asistencial completo y con enfoque cultural, centrado en el bienestar materno y recién nacido 2,3, en cuyo contexto resulta fundamental fortalecer estrategias orientadas a mejorar las intervenciones más efectivas a partir de la evidencia científica, lo cual incluye la creación y el desarrollo de espacios formativos que brinden a la mujer gestante y a su acompañante información clara, oportuna y pertinente, dirigida al desarrollo de habilidades para afrontar de manera segura los cambios físicos, emocionales y sociales propios del proceso gestacional, con el fin de favorecer una experiencia materna positiva 4.

En este sentido, el curso de maternidad y paternidad es un método de enseñanza, consolidado como una estrategia educativa, interactiva y estructurada, que si bien está diseñado para implementarse en el ámbito ambulatorio, donde las gestantes y sus parejas pueden acceder con mayor facilidad, puede incluir a mujeres hospitalizadas que por condiciones obstétricas requieren atención continua y no tienen acceso a esta formación, lo que limita su conocimiento y preparación para enfrentar el proceso reproductivo con conocimiento y seguridad. Trasladar esta estrategia al contexto hospitalario responde a una necesidad apremiante en términos de equidad y acceso, al permitir que estas mujeres también se beneficien de un proceso educativo integral, que fortalezca sus capacidades y promueva decisiones conscientes en torno a su salud sexual y reproductiva 2,7,8.

## METODOLOGÍA

Estudio de diseño cualitativo fenomenológico descriptivo, aplicado en la ciudad de Valledupar (Colombia), durante el periodo comprendido entre octubre y diciembre de 2024, y orientado a conocer las experiencias de las gestantes hospitalizadas con relación a la implementación del curso de maternidad y paternidad, con base en la fenomenología descrita por Husserl 9, la cual pretende generar datos descriptivos como discursos, emociones, sensaciones e intereses de los participantes 10, con el fin de explicar la esencia de la experiencia y describir las percepciones y las valoraciones de las mujeres involucradas.

La población de estudio estuvo conformada por 12 mujeres hospitalizadas en una institución de mediana complejidad que cumplieron con los criterios de inclusión: tener más de 18 años y una estancia hospitalaria superior a cinco días, a quienes se les impartió el curso de maternidad y paternidad en modalidad presencial intensiva, con una asistencia mínima a tres sesiones. Se priorizaron las necesidades individuales de las gestantes, teniendo en cuenta sus condiciones biológicas, como ser primigestantes o multigestantes, así como las complicaciones del embarazo por las que se encontraban hospitalizadas. La muestra fue construida a partir de los relatos de las gestantes, los cuales permitieron identificar conceptos clave y fundamentar las categorías empíricas (Tabla 1). Este proceso fue de carácter acumulativo y se desarrolló hasta alcanzar el punto de saturación teórica.

**Tabla 1:** Matriz de categorías

Objetivo general	Objetivo específicos	Categoría teórica	Subcategoría	Técnica
Conocer las experiencias de las gestantes hospitalizadas con relación a la implementación del curso de maternidad y paternidad	Caracterizar el perfil sociodemográfico de las gestantes y sus parejas	Condiciones sociodemográficas	Condiciones biológicas mujeres Edad Nivel de estudio Factor socioeconómico: Estrato Estado Civil Nacionalidad Paridad Edad gestacional Factor social Ocupación	Ficha de perfil sociodemográfico (encuesta tipo censo)
	Describir las experiencias de la población objeto de estudio con respecto al curso de maternidad y paternidad recibido	Recomendaciones de la OMS para los cuidados durante el parto, para una experiencia de parto positiva (OMS, 2018) Recomendaciones de la OMS sobre atención prenatal (OMS, 2016). Resolución 3280 (Colombia, Ministerio de Salud y Protección Social, 2018)	El curso como herramienta clave para la maternidad y la paternidad. Factores influyentes en la realización del curso. Percepción de la atención brindada durante el curso. Transformación del saber materno a partir de la educación recibida	Revisión sistematizada de la bibliografía y de artículos científicos Entrevistas semiestructuradas, relatos de situaciones vivenciadas en el curso de preparación para la maternidad y la paternidad

Las unidades de análisis incluyeron entrevistas en profundidad, narraciones y relatos, así como toda la información recogida directamente de las participantes 11.

Las categorías predeterminadas, alineadas con los objetivos del estudio, se organizaron en una matriz cualitativa construida a partir del marco conceptual y los antecedentes nacionales relacionados con el curso de preparación para la maternidad y la paternidad. La recolección de la información se llevó a cabo mediante la aplicación de una ficha sociodemográfica, con el fin de identificar las condiciones sociales, demográficas y biológicas más relevantes de las participantes. Las entrevistas semiestructuradas y en profundidad se realizaron por vía telefónica, debido a las dificultades de acceso a las residencias de las gestantes, ubicadas en zonas rurales dispersas y sectores con altos niveles de inseguridad. Al inicio de cada conversación se socializaron los objetivos del estudio, se solicitó el consentimiento informado y la autorización para grabar las conversaciones.

Las narraciones fueron posteriormente verificadas, contextualizadas e interpretadas por las investigadoras, quienes realizaron la transcripción textual, garantizando así los criterios de credibilidad, autenticidad y fidelidad 12 con respecto a las vivencias, los pensamientos, las emociones y las percepciones expresadas por las participantes en torno al curso.

### Análisis de datos

Se establecieron códigos para cada categoría y se asignaron pseudónimos a los participantes (Tabla 2). La información fue organizada en una matriz de análisis cualitativo que documentó tanto las categorías como sus subcategorías. Se identificaron patrones recurrentes considerados como datos de primer orden. Además, los resultados fueron sometidos a un proceso de validación interna y externa, así como a una estrategia de triangulación, que incluyó la comparación entre participantes, estudios previos y otras fuentes externas que evidenciaran experiencias sobre el curso de preparación para la maternidad y la paternidad, con el objetivo de garantizar la transferibilidad de los hallazgos.

**Tabla 2:** Perfil sociodemográfico

Código	Edad	Pseudónimo	Nacionalidad	Ocupación	Estrato	Nivel de estudio	Estado civil	Condición biológica	Diagnostico
PNC1	28	Mara	Colombiana	Estudiante	1	Decimo	Unión libre	Primigestante	26 semanas + IVU +Vaginosis
MNC1	34	Ana	Venezolana	Ama de casa	1	Bachiller	Unión libre	Multigestante	32,5 semanas + sífilis
PNC2	19	Paola	Colombiana	Ama de casa	2	Bachiller	Unión libre	Primigestante	31,1 semanas + HTA
PNC3	22	Maira	Colombiana	Ama de casa	1	Bachiller	Unión libre	Primigestante	30 semanas+ IVU
MNC2	28	Mirian	Colombiana	Ama de casa	2	Bachiller	Unión libre	Multigestante	33 semanas + IVU
PNC4	18	Astrid	Colombiana	Ama de casa	1	Bachiller	Unión libre	Primigestante	26 semanas + IVU +vaginosis
PNC5	18	Lucía	Colombiana	Ama de casa	2	Bachiller	Unión libre	Primigestante	22,5 semanas + IVU +ARO
MNC3	24	Carol	Colombiana	Ama de casa	1	Bachiller	Unión libre	Multigestante	25 semanas + síndrome febril
MNC4	38	Sandra	Colombiana	Ama de casa	3	Técnico	Unión libre	Multigestante	36 semanas + HTA
PNC6	24	Marta	Colombiana	Ama de casa	1	Técnico	Unión libre	Primigestante	30.4 semanas + vaginosis
PNC7	19	Silvana	Venezolana	Ama de casa	2	Bachiller	Unión libre	Primigestante	28 semana +

### Criterios de rigor

Los criterios utilizados para el análisis y la interpretación de los resultados en este estudio fueron la autenticidad, la credibilidad y la confirmación, garantizando que las investigadoras y los participantes se expresaran de manera auténtica, y que las descripciones se reflejaran con equilibrio, justicia y coherencia entre los relatos expresados por las mujeres sobre su experiencia durante el desarrollo del curso de maternidad y paternidad. Las investigadoras procuraron minimizar los sesgos en la interpretación de los datos, así como en sus reflexiones e ideas personales.

### Consideraciones éticas

Esta investigación fue aprobada en el marco de la convocatoria institucional de proyectos de investigación de la Universidad Popular del Cesar, lo que garantiza su pertinencia, así como su rigurosidad metodológica y ética. Fue clasificada como de riesgo mínimo, dado que la población objeto de estudio estuvo conformada por gestantes hospitalizadas en una institución de salud 13. A todas ellas se les brindó un trato respetuoso, asegurando la protección de la dignidad humana y de sus derechos fundamentales. La recolección y el procesamiento de la información se realizaron en cumplimiento de los principios éticos de confidencialidad, autonomía y consentimiento informado 14.

## RESULTADOS

El estudio contó con la participación de 12 gestantes hospitalizadas en una IPS de la ciudad de Valledupar, ocho de las cuales eran primigestantes, lo que hace especialmente significativa su participación, ya que el acceso a este tipo de información durante la hospitalización puede fortalecer su preparación para enfrentar con mayor seguridad los retos de la maternidad. La información sociodemográfica de las gestantes se detalla en la Tabla 3.

**Tabla 3:** Ficha sociodemográfica

Categoría	Datos
Edad	Entre 18 y 25 años: 8 gestantes Entre 26 y 35 años: 3 gestantes Entre 36 y 40 años: 1 gestante
Nacionalidad	Colombianas: 10 gestantes Venezolanas: 2 gestantes
Nivel de estudio	9: secundaria completa 1: secundaria incompleta 2: estudios técnicos
Estrato	Estrato I: 7 Estrato II: 4 Estrato III: 1
Estado civil	Unión libre: 12
Ocupación	Ama de casa: 11 Estudiante: 1
Condición biológica	Primigestante: 8 Multigestante: 4

### El curso como herramienta clave para la maternidad y paternidad

El curso brindó conocimientos fundamentales para enfrentar la maternidad, especialmente entre las primigestantes, quienes manifestaron que la intervención permitió aclarar dudas e inquietudes: "Yo era mamá primeriza y había muchas cosas que yo no sabía o sea era primera vez que yo estaba embarazada y me explicaron muchas cosas que yo no tenía conocimiento" (PNC-3) Maira. "Le sirve de mucho a las mamitas que están a punto de tener su niño, para que aclaren sus dudas y las que ya han tenido niño, pues aporten a las nuevas mamitas" (PNC-4) Astrid.

### Factores influyentes en la realización del curso

En los siguientes relatos las participantes expresan que, debido a la ubicación geográfica de su lugar de residencia, se les dificultaba asistir a las sesiones. Esta limitación evidenció una barrera para acceder a la información de manera oportuna: "Me gustaría también que estos cursos puedan llegar a zonas, como le digo, bastante alejadas, porque en mi pueblo no se ve eso, y si no hubiera sido porque hubiera llegado a Valledupar, pues, no me hubiera informado, no hubiera recibido las charlas; no hubiera aprendido tanto como ahora, entonces sería bueno que estos cursos pudieran llegar a zonas lejanas, como las mujeres de zona rurales, del campo para aprender de estos temas" (PNC-8) Carolina. "bueno, por lo menos si yo no hubiese estado hospitalizada en el momento, me imagino que no hubiera hecho el curso" (PNC-7) Silvana.

### Percepción de la atención brindada durante el curso

Los testimonios de las gestantes (PNC-7) y (MNC-2) evidencian que la atención recibida fue percibida de manera positiva, destacan las sesiones de los ejercicios y masajes, ya que les permitió relajarse y tener otra alternativa para manejar el dolor durante su embarazo: "Sentí que me ayudó bastante porque tú sabes que con el peso de la barriga uno le da dolor en las caderas y todo eso entonces sentía que con estos ejercicios sentía que me relajaba me sentía mejor" (PNC-7) Silvana. "Bueno, a mí me gustó de los cursos que asistí, donde ehh, estaba muy estresada en el hospital, me sentía mal, entonces hicieron unos... masajes, trajeron unas pelotas, me sentaron y me dijeron que me imaginara una parte donde hubiera tranquilidad y me sentí bastante bien de verdad, se me quitaron todas. lo que tenía, pues, tú sabes que a veces la emociones, como le digo, uno a veces se siente deprimida o sola en el momento, pero no porque quiera, sino porque toca. Pues entonces me sentí bastante bien" (MNC-2) Paola.

Asimismo, las participantes (PNC-2) y (MNC-1) valoran la información recibida sobre lactancia materna y parto humanizado, señalando que fue de gran ayuda para ellas: "Sí, fue información de calidad porque aprendí muchas cosas que no sabía, además me llamó mucho la atención sobre el parto humanizado, porque desconocía ese tema, no tenía ni idea de que existía el parto humanizado. y me pareció excelente", (PNC-2) Paola. "Sí, fue de buena calidad. Nunca había tenido una experiencia de poder orientarme sobre como el proceso del embarazo, la lactancia, entonces ahí aprendí mucho, una información de muy buena calidad" (MNC-1) Ana.

### Transformación del saber materno a partir de la educación recibida

Las participantes resaltan que la información recibida fue clave para fortalecer sus conocimientos sobre el proceso de maternidad: "El tema de poder obtener un acompañamiento durante el parto, eh, fue lo que más me motivó ya que, como ya llevo varios embarazos, este embarazo ha sido un poco complicado y ehh, eso me da un poco de tranquilidad al momento de yo dar a luz" (MNC-1) Ana. "Bueno me gustó mucho también la lactancia materna y la alimentación exclusiva porque bueno, las abuelas y también las mamás de uno siempre le han enseñado que a los niños uno debe hacerle las tomitas, debe hacerle la manzanilla para que bueno, supuestamente el estómago se les limpie, y todo eso, pero, ya aprendí que eso no es correcto, que eso no se debe hacerse con los recién nacidos." (PNC-6) Marta."Eh, a pesar de que yo tengo mis dos bebés no tenía conocimiento de cómo amamantar correctamente, como tiene que ser y pues en el curso aprendí cosas que no sabía y que no apliqué con mis dos bebés, porque no tenía conocimiento y eso, me gustaría compartirlo con alguien que no tenga conocimiento de esto" (PNC-7) Silvana.

## DISCUSIÓN

La preparación durante el embarazo es esencial para una transición efectiva hacia el rol materno. La educación proporcionada por profesionales de la salud, especialmente por el personal de enfermería, desempeña un papel fundamental al ofrecer orientación, apoyo y herramientas prácticas que fortalecen la confianza, las habilidades y las competencias necesarias para asumir las nuevas responsabilidades del cuidado. Los resultados muestran que para la mayoría de las participantes el curso representó una oportunidad significativa para afrontar sus miedos, adquirir herramientas útiles y, en el caso de mujeres multigestantes, corregir prácticas anteriores en el cuidado del recién nacido 15-17.

La hospitalización representa una limitación significativa para acceder a este tipo de acompañamiento, pese a que constituye una herramienta valiosa para reducir temores e inseguridades, fortalecer conocimientos y promover el autocuidado durante la gestación. Además, estas intervenciones contribuyen al ejercicio pleno de los derechos sexuales y reproductivos, los cuales con frecuencia son desconocidos por las gestantes en condiciones de vulnerabilidad.

Los conocimientos adecuados en las gestantes no solo potencian su autonomía, sino que también les permite desarrollar expectativas realistas y visualizar su maternidad con mayor seguridad, creando experiencias personales significativas. En este sentido, se hace necesario que el equipo de salud cuente con las competencias necesarias para orientar a las gestantes sobre los signos de alarma, cambios anatomofisiológicos, y demás elementos esenciales para fomentar el autocuidado, especialmente en mujeres primigestantes, lo que contribuye a disminuir la morbilidad y mortalidad materna. Así mismo se refleja en el estudio titulado Imaginarios sociales del curso de preparación para la maternidad y la paternidad en Colombia, donde un participante expresó 15,18: "Me parece que es un buen curso porque hay muchas mamás y papás que son primerizos, entonces no tienen ni la menor idea de cómo bañarlo, cómo cambiarlo, cómo cargarlo. entonces me parece bien" 15.

Los relatos de las participantes de este estudio demuestran lo funcional, eficaz y necesario que resulta la implementación del curso en contextos hospitalarios, reconocieron su importancia para la adaptación a los nuevos roles de la maternidad. Destacaron su valor en la erradicación de mitos, creencias y prácticas erróneas basadas en la desinformación. El impacto fue reportado como significativo, no solo en primigestantes o madres sin experiencia, sino también en mujeres con hijos que, sin haber accedido previamente al curso, desconocían aspectos fundamentales del cuidado del recién nacido y la lactancia materna, lo cual puede ser considerado un factor agravante de eventuales violencias obstétricas 19.

Muchas de las participantes que recibieron el curso hospitalizadas provenían de municipios y zonas rurales aledañas, por lo que si bien la mayoría manifestó que el curso cumplió e incluso superó sus expectativas, algunas gestantes señalaron la necesidad de ampliar su cobertura, haciéndolo más accesible para mujeres que, por condiciones sociodemográficas, tienen dificultades para asistir a un centro de salud dentro de la ciudad. Considerar esta opción es, sin lugar a dudas, una oportunidad para la promoción de hábitos saludables que benefician a las familias en su conjunto 20 ♠
